# Neural bases of sustained attention during naturalistic parent-infant interactions

**DOI:** 10.1038/s41598-025-22534-w

**Published:** 2025-10-31

**Authors:** Giulia Serino, Aleksandra A. W. Dopierala, Catalina Shen, Denis Mareschal, Gaia Scerif, Natasha Kirkham, Lauren L. Emberson

**Affiliations:** 1 Centre for Brain and Cognitive Development, School of Psychological Sciences, Birkbeck, University of London, London, WC1E7JL UK; 2 Baby Learning Lab, Department of Psychology, University of British Columbia, Vancouver, BC V6T 1Z4 Canada; 3 Department of Experimental Psychology, University of Oxford, Oxford, OX2 6GG UK

**Keywords:** Sustained attention, Infants, fNIRS, Naturalistic research, Attention, Human behaviour

## Abstract

Sustained attention—the ability to maintain focus on a particular location or object for an extended period of time—is a fundamental skill during development. It enables the developing child to acquire information about their environment, facilitating information processing and supporting memory. Despite its critical role in development, little is known about the neural mechanisms that support this ability. Building on insights from the ECG literature on sustained attention, the present study sought to address some of the challenges associated with neuroimaging research in developmental populations. Specifically, we developed and assessed the validity of a novel fNIRS protocol to study visual sustained attention within naturalistic contexts. Results indicated the involvement of left temporo-parietal areas during sustained attention in infancy. Furthermore, validation of the protocol demonstrated that different attentional states can effectively serve as baselines for studying specific components of attention. This new approach holds significant promise for future research aimed at extending the study of the neural substrates of attention to naturalistic settings.

## Introduction

The richness of the environment greatly influences how much attention an infant pays to a specific stimulus^1–4^. In this regard, it is important to take into account the complexity of this environment and its interaction with the developing brain to reach an in-depth understanding of attention. However, at present, little is known about how attention manifests in real-world or naturalistic infant experiences in general, and even less regarding how the brain supports such attentional manifestations^5^.

Traditionally, neuroscientific research has broken down complex behaviors into simpler components in controlled laboratory settings, aiming to understand the fundamental biological and physiological components underlying behavioral manifestations^6–9^. While this approach streamlines the investigation of cognitive phenomena and helps increase experimental control, it removes the intricate complexity and unpredictability inherent in real-world scenarios, the context within which attention operates and develops. An additional limit of experimental reductionism lies in its treatment of temporal dynamics. In laboratory settings, stimuli are often presented in a controlled, time-locked fashion, failing to capture the dynamic nature of real-world experiences^5,10^.This limitation is particularly pronounced in the study of attention, a phenomenon deeply rooted in time and context. Infants must constantly adapt their attentional resources to align with their immediate goals, the stimuli in their environment, and the constraints of their developing brains^11,12^. This delicate balance highlights the importance of studying attention and its rhythms as they naturally unfold in everyday infant experiences. Another critical consideration is that experimental tasks may not apply equally to all participants. A condition deemed “valid” for one individual may not effectively elicit selective attention for another participant, due to differences in developmental stage, cultural context, or individual variability. This variability, in turn, raises broader questions about the generalizability and replicability of experimental findings^13–15^. Increasingly, researchers are recognizing this limitation and exploring alternatives, such as observing behaviors as they naturally occur to better capture development in real-world settings^16–20^. Yet, opening the door to complexity does not come without a cost. Many are the challenges that neuroscientists are facing, such as the disparity between the observed behavior and the underlying brain activity across participants, the selection of an appropriate baseline, the high rate of movements, significant hardware and technical requirements, as well as extensive pre-processing time^21,22^.

The current study aimed to address some of these challenges, namely how to select an attention baseline in fNIRS studies conducted in real-world settings, and how to align behavioral observations with fNIRS recordings, to investigate how the brain supports sustained attention during infancy, within a naturalistic dyadic setting. It is the first study to use functional Near Infrared Spectroscopy (fNIRS)—a non-invasive neuroimaging technique that measures changes in blood oxygenation using near-infrared light, providing an indirect measure of neural activity^23^—to measure localized hemodynamic changes in the infant brain during these naturalistic interactions and to characterize the neural bases of sustained attention in complex real-world settings.

### Sustained attention

The emergence of sustained attention plays a pivotal role in cognitive development. It enhances various cognitive processes, such as recognition memory, as well as the ability to inhibit distractors, favouring the processing of relevant information^24–28^. Here, we specifically focus on visual sustained attention: the ability to concentrate on a given visual stimulus to enhance information processing^29–32^. This ability emerges around the third month of age, corresponding to when the young infant can maintain an alert state during much of the daytime hours; however, at this point, it heavily depends on externally presented stimuli, generally provided by the caregiver^9,33^. The capacity to a*ctively* engage with and focus on stimuli in the environment emerges around the fifth month of age^24,32,34^, possibly due to the concomitant development of frontal circuits, gradually increasing through the second and third year of life^24,35^.

### Measuring sustained attention in everyday infant experiences

Sustained attention in infancy has been predominately studied using behavioral assessment (e.g., eye movements), heart rate fluctuations, and electroencephalography (EEG)^31,32,36,37^.

The behavioral (and neuroscientific) traditions are based on the assumption that attention moves with the eyes and thus use eye behavior as one of the primary markers of sustained attention^31,32,36–39^. As such, infants’ looking time is often measured in behavioral assessments. One of the benefits of behavioral assessment is that attention can be measured by coding infants’ behavior in a naturalistic environment. However, despite numerous studies on the subject^32,37^, there remains a lack of consensus on how to best measure infants’ sustained attention. Studies suggest that the minimum looking time required to identify episodes of attention generally ranges from 1 second^10,40^ to 3 seconds^41^. To enhance the robustness of these assessments, researchers often pair eye gaze measurements with concurrent observations of behaviors such as object manipulation or soothing actions^42–44^. However, variability in how sustained attention is conceptualized and operationalized, combined with the inherent subjectivity of manual coding, underscores the critical need for standardized protocols to improve the consistency and reliability of behavioral assessments of sustained attention.

**Fig. 1:**
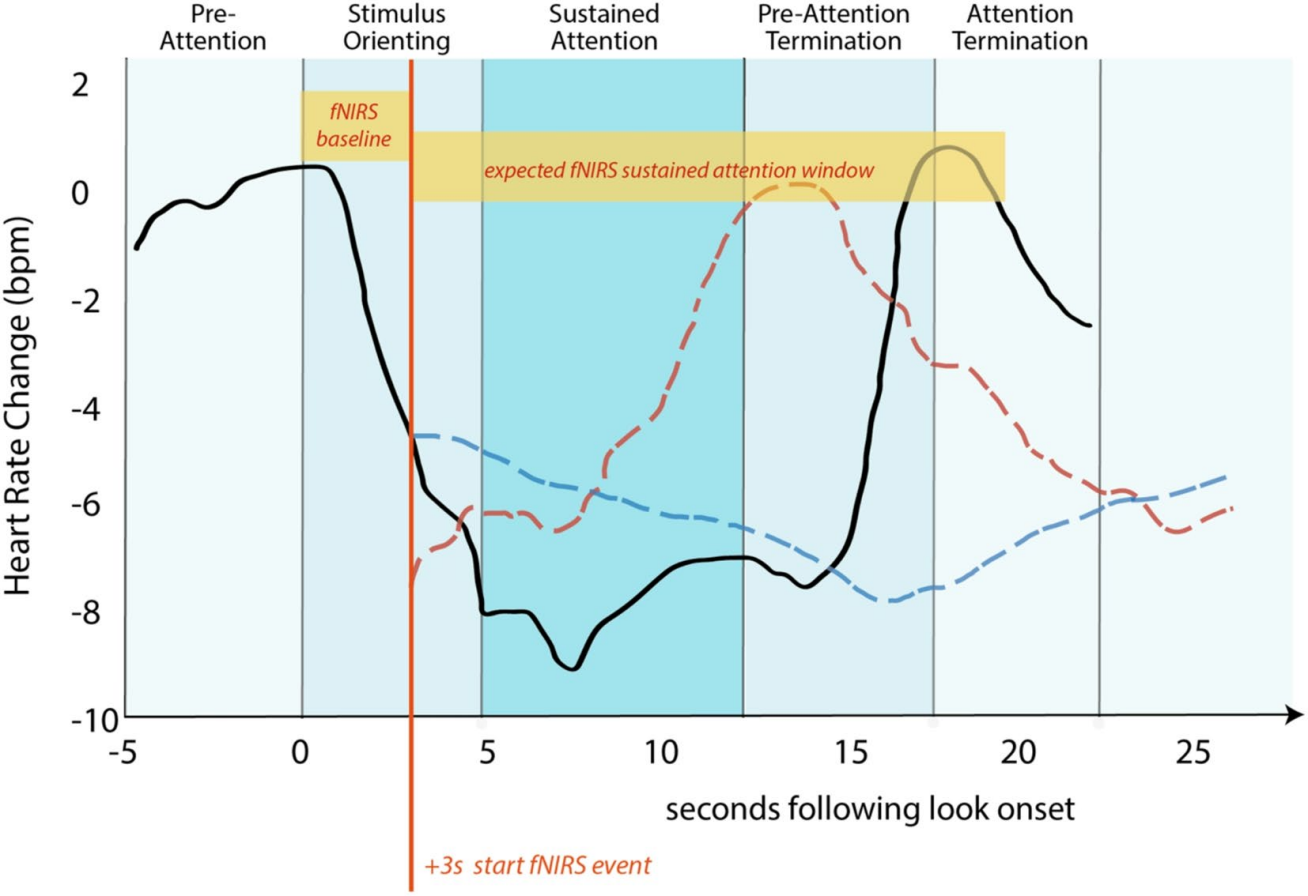
Expected heart rate and fNIRS hemodynamic changes during visual sustained attention. The solid black line represents heart rate changes during sustained attention, as identified by Richards and Casey (1991). The dotted lines represent the expected timing of the hemodynamic response related to sustained attention, as captured by the fNIRS signal, accounting for its typical delay—red for HbO and blue for HbR. The vertical red line indicates the (delayed) + 3 s onset of the fNIRS sustained attention blocks. Heart rate changes and hemodynamic responses are shown for illustrative purposes only and were adapted from Richards and Casey (1991) and Issard and Gervain (2018), respectively.

Heart rate fluctuations are more objective compared to behavioral observations, as they are not subject to coder bias. As such heart rate data are often combined with behavioral observations to enhance the robustness of attention assessments^34,42^. Specifically, heart rate studies use electrocardiography (ECG) to examine heart rate patterns and identify different attention phases while the infant engages with a target. These phases include attention orienting, sustained attention, and attention termination^26,34,45,46^. Orienting to a target typically induces a deceleration in heart rate compared to the baseline (i.e., no target). This deceleration is followed by a plateau in which the heart rate remains slower compared to the baseline, typically lasting between 5 and 15 s after stimulus onset. This time interval corresponds to the sustained attention phase and usually coincides with the baby being focused on the target at a behavioral level^42^. The heart rate then returns to baseline during the attention termination phase (Fig. 1).

The neurophysiological markers associated with sustained attention have predominantly been investigated using event-related potentials (ERPs) and electroencephalography (EEG) in traditional laboratory settings^47^. The ERP literature has reported a negative component (Nc) during attention tasks, but this appears to be less specific to sustained attention and more broadly associated with attention mechanisms, including attentional orienting and increased arousal^47–49^. In EEG studies, higher theta power has been consistently found during sustained attention, along with alpha attenuation^28,50,51^. Applying cortical source analysis to EEG oscillations, Xie and colleagues^52^ recently reported that theta synchronization during sustained attention was primarily localized in the orbitofrontal lobe, temporal pole, and ventral temporal lobe.

All these studies have provided valuable insights into the neurophysiological markers of sustained attention in laboratory settings^28,47,49–52^. However, EEG techniques have a low tolerance for movement. Consequently, EEG studies typically rely on researcher-led stimulus presentations rather than capturing infant-directed attention (although see^33^), which limits their generalizability to naturalistic settings. Additionally, while EEG and ERP provide optimal temporal resolution, they offer limited spatial localization^53^. This limitation underscores the importance of employing neuroimaging techniques, such as fNIRS and fMRI, to study the neural basis of attention. If we look at adult literature, imaging studies have provided significant insights that have advanced our understanding of attention. For example, foundational work by Posner and Petersen^54^ on the neural underpinnings of attention transformed the conceptualization of attention from a unitary mechanism to a network comprising distinct subcomponents, each supported by specific neural systems. Building on this, Corbetta and colleagues^55,56^ characterize the neural substrates of attention orienting thanks to a meta-analyses of fMRI studies. Turning to sustained attention, in their meta-analysis of 67 fMRI studies, Langner and Eickhoff^57^ identified 14 clusters that were consistently activated across various sustained attention tasks, including both subcortical regions (e.g., thalamus and cerebellum) and cortical areas in the frontal, premotor, and parietal lobes. These findings highlight the complexity of attention and the need for neuroimaging methods to capture its multifaceted nature. 

Nonetheless, evidence from infant neuroimaging studies remains limited and requires investigation. Examining the neural correlates of attention in early development is critical not only for understanding the infant brain, but also for elucidating developmental trajectories. Cognitive skills are not fixed traits confined to a particular developmental stage; rather, they emerge as dynamic outcomes of ongoing interactions between brain maturation and environmental inputs^58^. Adopting a developmental perspective in the study of attention is essential for understanding cognitive mechanisms in both adults and children, as domain-specific outcomes emerge through gradual developmental processes unfolding over ontogenetic time^58–63^.

A few brain imaging studies^64–66^ have reported frontal involvement and some degree of temporo-parietal engagement during lab-based attention tasks with infants. However, none have specifically addressed the neural mechanisms of sustained attention in infancy. This gap likely arises from the limitations of current brain imaging techniques for studying infants, such as limited temporal resolution and related challenges in isolating attention-related brain activity.

The primary objective of the current study was to design a protocol to address some of these challenges and extend the study of the neural bases of infant attention to naturalistic settings. Specifically, we aimed to investigate the neural bases of sustained attention in infants as attention unfolds naturally, without imposing any artificial constraints on its temporal dynamics or infant agency. Within this project, parents were invited to engage in play sessions with their infants, replicating everyday toy-play experiences at home. Infants’ brain activity was measured with fNIRS for the entire duration of the sessions. The present study was part of a broader research project aimed at investigating brain development and object exploration during naturalistic interactions in the first year of life^16^.

FNIRS was chosen to investigate brain activity in naturalistic contexts, given its good spatial resolution, robustness to motion, and suitability for use in infant research^16,53^. FNIRS equipment is lightweight, wearable for extended periods, and relatively robust to motion, allowing participants to engage in unstructured, naturalistic interactions. However, similarly to other neuroimaging techniques, fNIRS relies on the assessment of stimulus-evoked hemodynamic changes relative to a baseline condition^22,67,68^. While this approach is generally effective, it can pose a specific challenge in the study of attention, as attention, in its various forms, is continuously present during wakefulness, complicating the definition and use of baseline conditions. To address this limitation, we leveraged distinct attention states to isolate brain activity uniquely associated with sustained attention. Specifically, we applied the temporal dynamics of attention, as identified in earlier studies using ECG^26,34,45,46^, to guide the identification and extraction of sustained attention during play, at both behavioral and neural levels. The validity of our experimental protocol was assessed by systematically adjusting (e.g., postponing or anticipating) the onset of these attention phases and evaluating the corresponding changes in the temporal unfolding of the hemodynamic response, and by using statistical methods to approximate randomness (e.g., permutation testing). Three regions of interest (ROIs) encompassing frontal and temporoparietal areas—brain regions previously implicated in attention processes in both infants^64–66^ and adults^69,70^ —were defined. Hemodynamic changes within these regions were analyzed to investigate the neural mechanisms of sustained attention in infancy.

## Results

### Behavioral finding

Average looking time to stimuli in the room (e.g., toy, caregiver, etc.) across participants ranged from M = 1.84 s (SD = 1.1.6) to M = 5.69 s (SD = 5.65). In line with the experimental design, participants looked longer and more frequently to toys than any other targets in the room (Table S1). Similarly, when considering only sustained attention events (i.e., looking time > 8 s), the majority of observations were focused on toys (N = 124), with a few observations focused on the mother (N = 3) and the mother’s body (N = 7). Due to the small number of occurrences of long observations towards non-toy targets, only toy observations were selected for subsequent fNIRS analysis. A threshold of a minimum of 5 observations per participant was set to ensure an adequate number of events for the subsequent fNIRS analysis. All participants met this criterion and were included in the analysis (Table S2).

### Neural correlates of sustained attention: fNIRS findings

Raw fNIRS data were pre-processed in HOMER2^71^, following the guidelines by Di Lorenzo, Pirazzoli et al.^72^ [set 3]. Channels with raw intensities below 2 µM or above 10 µM, or those with excessive motion artifacts (SDrange [0–45]), were excluded. Raw data were then converted to optical density and corrected for motion artifacts applying wavelet analyses^72^ (iqr = 0.8) and spline correction^73,74^ (p = 0.99). A low-pass filter at 1 Hz and a high-pass filter at 0.01 Hz were applied to remove physiological noise. Data were converted to relative concentrations of HbO and HbR with the modified Beer-Lambert law [DPF = 5.1, 5.1]. Subsequently, data were averaged into 18-s blocks: a 3-s pre-stimulus baseline beginning 3 s before the delayed onset of a sustained attention block, and a 15-s post-stimulus block. For each participant, peak concentration of HbO and HbR were determined channel by channel, based on maximum or minimum values within the defined activation window. A time period of ± 2.5 s around each peak was extracted and averaged to compute the mean concentrations of HbO and HbR^75,76^. Multiple t-tests were then conducted to analyse the mean concentrations of HbO and HbR in the ROI analysis and across channels. False discovery rates (FDR) were employed to adjust *p* values for multiple comparisons using the R package qvalue^77^.

Based on previous evidence^64–66,70^, three anatomical regions of interest (ROIs) were delineated bilaterally (Table S3). Specifically, the devfOLD’s LPBA40 atlas^78^ [age 6 months] was used to identify channels above the medio-frontal gyrus [MFG] (2 channels), the superior temporal gyrus and sulcus [STS] (6 channels), and the temporo-parietal junction [TPJ], including two channels above the angular gyrus and one above the supramarginal gyrus (Figure S2).

One-tailed t-tests were conducted for each ROI individually to assess the increase in HbO during sustained attention blocks. Results showed increased activity in the left STS (*t*(10) = 2.633, *p* = 0.024, *pFDR* = 0.02) and left TPJ ROIs (*t*(10) = 2.213, *p* = 0.026, *pFDR* = 0.02) (Table 1). T-test comparisons were conducted to evaluate changes in the mean concentration of HbR in each ROI. No significant change in activity was found in this chromophore (please refer to Table S5 in supplementary materials for further details).

**Table 1:** Results one-tailed t-test (+ 3 start) for mean HbO concentrations during sustained attention for each ROI*.*

ROI	M	SD	*t*	*df*	*p*	*q*	*d*	*power*
lMFG	1.941e−02	3.033e−01	0.222	11	0.414	0.115	0.06	0.08
lSTS	2.882e−01	4.224e−01	2.263	10	**0.024**	0.021*	0.68	0.72
lTPJ	3.181e−01	4.767e−01	2.213	10	**0.026**	0.021*	0.67	0.70
rMFG	1.204e−01	5.961e−01	0.670	10	0.259	0.087	0.20	0.16
rSTS	1.593e−01	3.784e−01	1.396	10	0.096	0.054	0.42	0.39
rTPJ	1.009e−01	4.359e−01	0.768	10	0.230	0.087	0.23	0.19

L = Left; R = Right; MFG = Medial Frontal Gyrus; STS = Superior Temporal Sulcus; TPJ = Temporoparietal Junction; M = mean (unit measure: Mol/L); SD = standard deviation; t = t-value; df = degrees of freedom; p = * p* values; q = * p* values s corrected for multiple comparisons using the False Discovery Rate (FDR) method via the R package qvalue (Storey, 2024); d = Cohen’s d. Power was computed with the R package pwr (Champely, 2020) at the conventional α = 0.05 level to estimate test sensitivity.

*Significant values are in bold.

Channel-by-channel analysis indicated a significant increase in HbO in a group of channels located above the temporoparietal areas, both in the left hemisphere (ch8: t(10) = 2.23, *p* = 0.02; ch9: t(10) = 2.55, *p* = 0.01; ch10: t(10) = 2.02, *p* = 0.04) and the right hemisphere (ch30: t(10) = 2.03, *p* = 0.03; ch32: t(11) = 1.80, *p* = 0.05). However, none of these *p* values survived FDR correction for multiple comparisons. Similarly, t-test comparisons show a decrease in mean concentration of HbR in ch32 (t(10) = − 3.67 , *p* = 0.004) and ch21 t(10) =  − 2.19, *p* = 0.05), but none of the *p* values survived FDR correction for multiple comparisons (please refer to Table S8 and Table S9 in supplementary materials for further details).

To further characterize the spatial distribution of sustained-attention–related brain activity and control for multiple comparisons across channels, a cluster-based permutation analysis was performed on the HbO data. One-sample t-tests were conducted at each channel, with a cluster-forming threshold set at t > 2.0. Channels exceeding this threshold and spatially adjacent were grouped into clusters. The cluster mass was calculated as the sum of t-values within each cluster. To assess statistical significance, a nonparametric permutation procedure with 1000 iterations was applied. In each iteration, subject-level data were randomly sign-flipped to simulate the null hypothesis, and the maximum cluster mass was recorded to construct a null distribution. The observed cluster spanning Channels 8–10 yielded a significant cluster-level *p* value of 0.002. This cluster overlapped with the left temporoparietal junction (TPJ) and left superior temporal sulcus (STS) ROIs, reinforcing the spatial specificity of the ROI-based findings. Full methodological details and extended results are available in the Supplementary Materials.

### Protocol validation

To evaluate the efficacy of the fNIRS protocol to study sustained attention, ROI- and channel- wise analyses were repeated anticipating and postponing the start times of the sustained attention blocks and temporal unfolding of the hemodynamic response (HR) within each channel was inspected. First, the start of each sustained attention block was aligned to zero (*start* + *0*), synchronizing it with the actual onset of object-directed looking behavior. Data pre-processing and statistical analysis remained consistent with prior procedures (*start* + *3*). The sole modification was setting the baseline to the 3 s preceding the observed looking behavior. One-tailed t-tests revealed no significant increase in HbO concentrations across any ROIs or channels (Table S6, Table S10, and Table S11). Nevertheless, the shape of the HR provided valuable insights into the accuracy of the experimental design. Visual inspection of the HR at + 3 and + 0 start times (Figure S3) showed that aligning event onset to zero resulted in HR peaks occurring towards the end of the sustained blocks, with the increase in HbO around the peak potentially extending beyond the analyzed block duration in some participants. In line with previous ECG evidence^46^, this supports the hypothesis that infants transition into sustained attention a few seconds after initial engagement with a stimulus and may help explain the lack of significant findings with a + 0 start.

The same analysis was repeated by postponing the start time of each event to 6 seconds (+6 start) into the the object-directed looking behavior (Table 7, Table S12, and Table S13). Channels 9 (t(10) = 3.126, *p* = 0.005), 30 (t(10) = 1.825, *p* = 0.05), and 32 (t(10) = 1.815, *p* = 0.05) remained significant. Additionally, Channels 6 (t(10) = 1.957, *p* = 0.04) showed a significant increase in HbO. However, none of these results survived FDR correction for multiple comparisons. As predicted, visual inspection of the HR shows that the + 6 s start effectively captured the peak of the hypothesized sustained attention window (Figure S3). However, the + 3 s start likely represents the optimal balance between study feasibility, given infants’ attentional capacities, and the likelihood of capturing the sustained attention window in the fNIRS signal. Although behavioral data indicated that infants could sustain attention for approximately 13 s on average, there was considerable variability across subjects (Table S2). This variability likely contributed to increased noise in the + 6 s baseline condition compared to the + 3 s condition (please refer to Table S4 and S7 in supplementary materials for further details).

Lastly, to ensure that our findings were not driven by specific window definitions, an additional analysis was conducted by randomizing the start of each attention window (± 15–30 s). In line with our hypotheses, this random sampling of the fNIRS recordings yielded no significant activity at either the ROI or channel level.

Although these complementary analyses enhanced the robustness of our findings and the protocol validation, future studies should incorporate direct heart rate recordings or extract heart rate information from the fNIRS signal^79^ to systematically assess the overlap between heart rate fluctuations and fNIRS activity. Unfortunately, the sampling rate of the fNIRS device used in the current study (5 Hz) was too low to reliably capture heart rate variability.

## Discussion

This study aimed to develop an fNIRS protocol to investigate brain activity associated with sustained attention during naturalistic interactions. To achieve this, infant-caregiver pairs were invited to play with toys while fNIRS measured infants’ brain activity. Instances where infants engaged with an object for more than 8 s were identified through video-coding analysis. Temporal patterns of attention, as identified in previous studies using ECG^26,34,45,46^, were applied to segment these observations into pre-attention, attention orienting, and sustained attention phases, which were then linked to the fNIRS data. These distinct attention phases were leveraged to establish the fNIRS baseline and isolate brain activity specifically linked to sustained attention. Specifically, fNIRS activity corresponding to sustained attention were analyzed starting 3 s after the onset of a look, with the baseline defined as the 3 s preceding it. Results revealed an increase in HbO in brain regions surrounding the posterior part of the left superior temporal gyrus, the supramarginal gyrus, and the angular gyrus. Consistent with the ROI analysis, the cluster-based permutation analysis showed increased HbO in three channels above the temporoparietal areas in the left hemisphere. Similarly, the channel-by-channel analysis showed increased HbO around these regions, although this effect did not survive FDR correction. The observed increase in brain activity in adjacent channels in regions previously associated with attention in both infants^64,65^ and adults^69,70^, together with the canonical shape of the HR, characterized by a peak falling within the expected sustained attention window, suggest that the increase in HbO can be attributed to sustained attention, substantiating the experimental validity of the experimental protocol.

Furthermore, our findings demonstrate the suitability of fNIRS as a reliable tool for studying infant populations in naturalistic research settings. The fNIRS attrition rate in our study was comparable to standard rates reported in the field and consistent with those observed in more controlled laboratory settings^13^. Despite variability in average looking times, all infants exhibited a sufficient number of sustained attention events to be included in the analysis, with minimal attrition due to motion artifacts.

Another contribution of the current study is the demonstration that leveraging different attentional states can effectively simulate the contrast between experimental and baseline conditions commonly employed in fNIRS paradigms. Selecting an appropriate baseline in naturalistic studies is an area of active debate in the fNIRS literature^22^. An effective baseline should refrain from engaging brain regions of interest while accounting for low-level factors that might bias the results, such as motion or physiological noise. However, this task becomes particularly challenging when studying attention, as attention, in various forms, is continuously present during wakefulness. In this context, rather than attempting to identify moments with no attention, we leveraged different attentional states to define an appropriate baseline. Specifically, we selected moments when the infant oriented towards an object (corresponding to the pre-attention and orienting phases in attention ECG studies^46^) as an active baseline^22,80–82^ for the subsequent sustained attention phase and shifted the start of the sustained attention blocks by 3 s when aligning behavioural and fNIRS data. Since increases in HbO are typically detectable 5–8 s following stimulus presentation^83^, shifting the start of the sustained attention blocks into the look enabled us to reduce the likelihood of capturing brain activity related to the preceding attention orienting phase while maximizing the likelihood of capturing brain activity related to sustained attention.

Despite these contributions, this design also presents some limitations. First, while our findings suggest that temporal-parietal areas are involved in sustained attention during naturalistic parent-infant interactions, it is important to acknowledge that temporoparietal areas have also been implicated in object exploration^84,85^ and social processing^82,86–89^. It is therefore possible that the observed increase in brain activity in these regions is due to interactions between infants and caregivers rather than attentional processes. We deem this unlikely, as caregivers were not instructed to interact or speak with the infant exclusively during specific time windows (i.e., the sustained attention block vs the preceding baseline). However, this aspect was not systematically controlled for, and future studies are needed to address this possibility. Similarly, a larger dataset including non-toy events will help to further confirm that the detected activity within the selected time window is linked to sustained attention rather than solely object processing.

Furthermore, in contrast to previous evidence^64–66,70^, no significant activity was observed in the frontal areas; and activation in the STS and TPJ was detected only in the left hemisphere, with no corresponding activation in the right hemisphere. This discrepancy may be attributed to the fact that the baseline condition —pre-attention state and attention orienting—potentially involved right hemisphere activity, which was cancelled out during baseline correction. This interpretation is consistent with the involvement of frontal areas and the right temporo-parietal junction (TPJ) during attention orienting^65^. This outcome highlights a potential pitfall of using one behavior as a control baseline for another. While leveraging distinct attention phases offers substantial advantages—such as avoiding constraints on infants’ behavior and enabling the study of attention in naturalistic settings—it also poses the risk of canceling out activity in regions engaged during both processes (i.e., sustained attention and attention orienting). On a related note, behavioral differences corresponding to changes in attentional state could act as confounds when using one attentional state as the baseline for another. For instance, orienting attention towards an object typically involves a higher frequency of eye and head movements compared to sustained attention periods, potentially introducing noise into the data. Here, this issue was addressed by two factors: (i) brain activity over the motor cortex and superior eye fields, regions strongly associated with eye movements^90,91^, was not included in the subsequent analysis, and (ii) head and eye movements also occur during sustained attention periods^92,93^, reducing their specificity as confounding factors.

Lastly, it is important to note that the fNIRS array was designed for a larger project aimed at investigating brain development during naturalistic interactions^16^ and not specifically to target brain areas involved in attention. Although attempts were made to cover these areas as comprehensively as possible with the selected ROIs, it is conceivable that brain activity related to sustained attention extended into the parietal and frontal regions, and the current fNIRS array did not enable us to detect it. Furthermore, due to limitations imposed by the fNIRS array, the number of channels within each ROI was not balanced. The STS ROIs were larger than that the MFG and the TPJ ROIs. This imbalance resulted in over and underrepresentation of certain brain areas, introducing noise into the subsequent statistical analysis. In addition, it is important to acknowledge that, given the limited sample size (N = 12), the findings should be interpreted with caution. A post hoc power analysis for the main ROI findings indicated statistical power above 0.70 for both the left TPJ and left STS, suggesting moderate sensitivity to detect effects in these regions^94^. However, these power estimates are based on the sample size and the number of observations (blocks) per participant. The number of blocks was relatively high in our study (124 in total), possibly enhancing statistical sensitivity^95^. As such, the estimates reflect observed effect sizes but do not fully address concerns about the robustness and generalizability of the result^96,97^. Replication with a larger sample is therefore necessary to reduce the risk of false-positive findings.

To conclude, this study is the first to examine the neural correlates of infants’ sustained attention as attention naturally unfolds, without imposing constraints on its rhythms. Our findings highlight the activation of left temporo-parietal areas during sustained attention, shedding light on the neural mechanisms underlying this critical developmental process. In addition, our results demonstrate that manipulating event timelines during naturalistic interactions can effectively simulate the contrast between experimental and baseline conditions, typically used in fNIRS paradigms. By segmenting data to align with naturally occurring behaviors, this approach enables the isolation of distinct attentional states and their associated neural underpinnings without imposing restrictions on infants’ spontaneous actions. This novel approach offers a valuable tool for exploring the neural correlates of attention, paving the way for future research into the dynamics of attention and cognition in real-world settings, as well as the developmental trajectory of attention networks from infancy. Furthermore, our findings underscore the suitability of fNIRS as a reliable method for naturalistic research with infant populations.

Future studies might consider integrating both EEG and fNIRS to gain a more comprehensive understanding of the mechanisms underlying sustained attention at both temporal and spatial levels. While fNIRS is well-suited to naturalistic settings and offers good spatial resolution, it provides limited temporal resolution. Combining it with EEG could strengthen fNIRS findings: if localized fNIRS activity is consistently associated with temporally precise EEG signals evoked by attention episodes at the behavioral level, this would provide stronger evidence that the hemodynamic changes measured by fNIRS are related to attentional processes. Moreover, this multimodal approach would allow for a more precise understanding of the spatial and temporal dynamics of attention. In addition, while our current approach was effective at the group level, it lacked sensitivity to individual differences in sustained attention. Future research could address this by combining ECG and fNIRS to better capture these interindividual differences. ECG data could inform the selection of attention windows^52^, enabling a more accurate mapping of variability in brain activity, as measured by fNIRS, to fluctuations in sustained attention.

## Methods

### Participants

Nineteen healthy full-term infants participated in the study, (13 females, 6 males; M = 5.91 month, SD = 0.78). Ethnicity and family culture were reported by parents through a standardized questionnaire with predefined categories and an open-ended option for additional backgrounds. The reported ethnic composition included European (n = 4), East and Southeast Asian (n = 2), South Asian (n = 1), West Asian (n = 1), and mixed backgrounds such as European and Australian (n = 1), Chinese and Iranian (n = 1), European and East Asian (Japanese) (n = 1), and Pacific Islander and South Asian (n = 1). Family cultural backgrounds reported by parents included European, Canadian/USA, Chinese, Australian, West Asian, East and Southeast Asian, and South Asian.

A subgroup of 7 infants was excluded from data analysis because they did not reach the minimum duration criteria of 5 minutes of fNIRS data (n = 3), incorrect cap placement (n = 1), or because of poor optode-scalp in more than 50% of the channels (n = 3) after running through the QT-NIRS toolbox (Quality Testing of NIRS^98^). This resulted in a final sample of 12 infants (9 females, 3 males; M = 5.86 month, SD = 0.75), contributing to high-quality fNIRS data. The study was approved by the ethics committee of University of British Columbia and performed in accordance with the Declaration of Helsinki. Informed consent was obtained from all participants and/or their legal guardians.

### Design and procedure

Infants were tested in a quiet room at the Baby Learning Lab of the University of British Columbia. The room was equipped with a floor pad and three video cameras located on the sides of participants to record participants’ behavior during the study. A curtain separated the researchers and the family during the study. The study took place during the COVID-19 pandemic. Due to COVID restrictions, caregivers and researchers were asked to wear a mask for the entire duration of the visit. Before their visit, caregivers were instructed to bring three or more toys that their infants typically play with that were not electronic and preferably that infants could hold in their hands. Upon arrival, infants and caregivers were welcomed into the testing room and invited to seat on floor pad with their infant. Researchers and caregivers then selected three toys from those brought by the caregiver. Subsequently, the caregiver was presented with an additional six baby toys (supplementary materials, Figure S1) and asked to choose three that were unfamiliar to the infant and that they thought the infants would be interested to play with. All the selected toys were then placed in a box, and the box placed close to the caregiver to ensure easy reaching of the toys during the study. We instructed the parent to only take out one toy at a time, so that the rest of the toys remained in the box, out of infant’s sight.

Once the fNIRS cap was placed on the baby’s head and photos/videos were taken of the infant in the cap for cap location verification (see more details below), the caregiver was invited to sit comfortably in a position facing the infant. A U-shaped nursing pillow was provided for added support to the infant. This positioning allowed the caregiver to interact freely with the baby and play with the toys. Caregivers were instructed not to bounce the infant and reduce large infant movements to avoid the fNIRS cap moving and only play with one toy at a time. Otherwise, they were encouraged to play as they would at home with their infant. When comfortable, the caregiver was invited to start playing with the baby, using the toys from the box at their pleasure. The testing session continued until the baby became fussy or 20 min elapsed. The 20-min limit was chosen to find a balance between maximizing fNIRS data collection, ensuring the baby’s comfort, and avoiding excessive post-testing video-coding workload.

### fNIRS array and cap placement

fNIRS data were recorded using a NIRSsport2 system (NIRx Medical Technologies, NY, USA BV, Netherlands), with a custom design array covering the occipital, frontal, and temporo-parietal areas bilaterally. The array was designed as part of broader projects aimed to investigate brain activity in naturalistic interaction^16^. Each device had 8 light sources and 8 detectors, resulting in 22 channels per device and a total of 44 channels. The source-detector (S-D) separation was approximately 20 mm. Two continuous wavelengths of near-infrared light (763 nm and 841 nm) were utilized to measure changes in HbO and HbR concentration. The sampling rate was set to 5 Hz. The optodes were fitted into an EasyCap. The cap size used was either 42 cm (9 participants), 44 cm (2 participants), or 46 cm (1 participant), depending on the participants’ head size. Cap placement during data collection was carefully documented by capturing photographs of each participant’s head from multiple viewpoints. Special attention was given to ensuring proper cap alignment, with the front edge of the cap positioned precisely above the participant’s eyebrows. To maintain data quality, the photographs were reviewed prior to data analysis by a trained researcher. Cap placement was evaluated for significant misalignment. If a cap was judged to be improperly positioned, the corresponding participant’s data was excluded from analysis (N = 1).

### Operationalization and extraction of sustained attention events from behavioral observations and fNIRS data

**Fig. 2:**
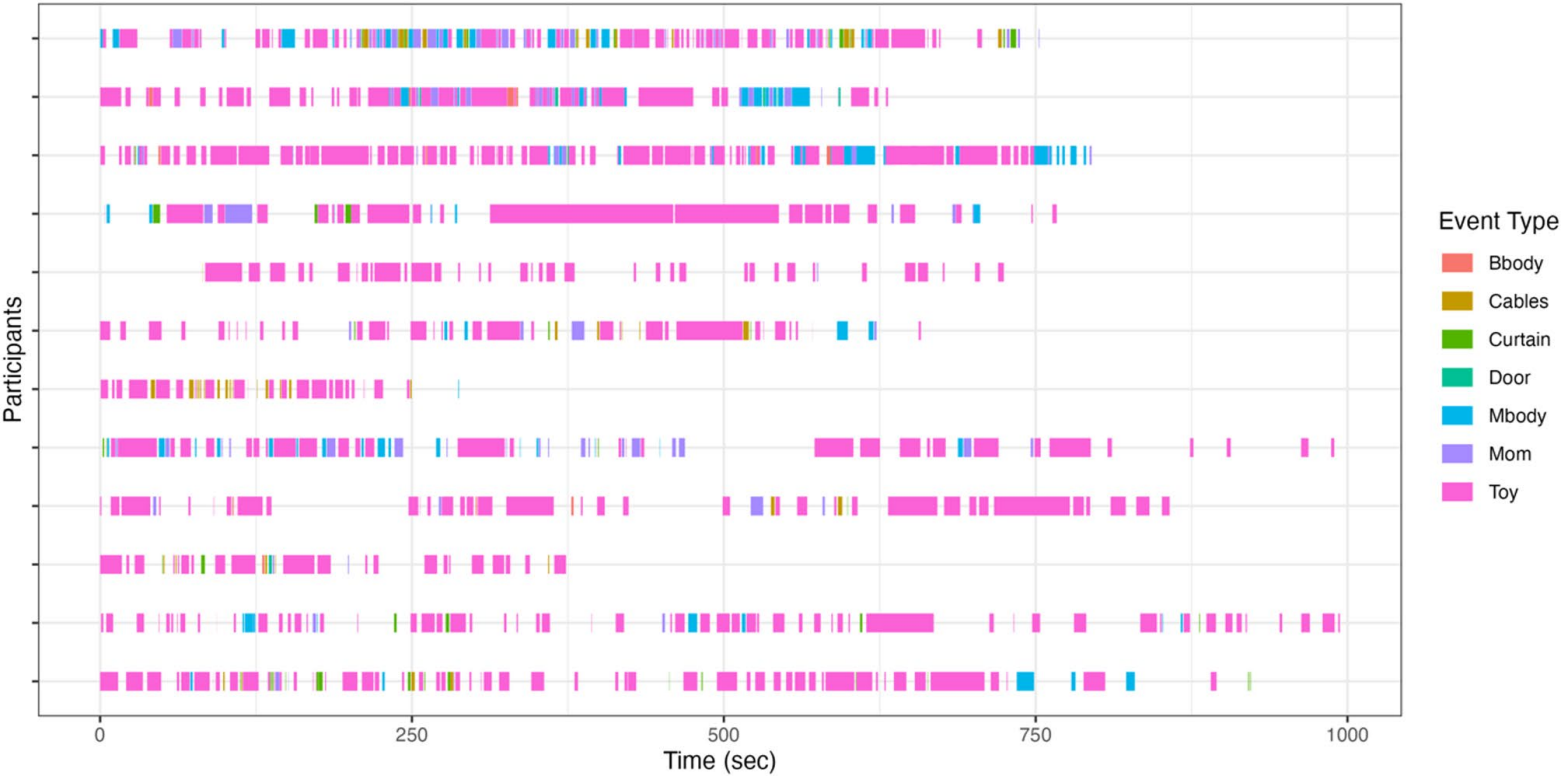
Extract of behavioral video-coding. Each line represents a participant. Time 0 represents the start of the toy-play session between participant and the caregiver. Bbody = infant’s body; Mbody = Caregiver’s body; Mom = Caregiver’s face; Cables = fNIRS device cables; Curtain = curtain separating the family from the testers; Door = room door; Toy = toy object.

To ensure high reliability, video-coding was conducted in ELAN 6.4 (https://archive.mpi.nl/tla/elan) by two independent coders. Each coder scored half of the sample. The other half was reviewed by the second coder. Both coders watched all the videos; instances of coding disagreement were underlined and subsequently discussed between the coders to reach a consensus. The goal of the video coding was to determine time-points where infants were clearly looking to a singular object or set of objects. All the events in which the infant looked at an object and/or person, as indicated by their eyes and the head clearly oriented to the object, and the baby’s hands and mouth either interacting with the object or remaining still and not engaging with a different object were identified and coded as a bout of attention and the target of that attention was noted (Fig. 2). All possible objects or targets were considered, including the toys, caregiver, curtains, door, fNIRS cables, the infant’s body, and the caregiver’s body. The observations were ended when infants looked away from the object/target for more than 100 ms. This time interval was chosen to capture shifts in attention orienting as saccadic latencies typically take around 100 ms to 500 ms to be planned^99–101^. While it is possible that other shifts of attention were not captured by this coding scheme, the temporal resolution of fNIRS does not allow for the detection of rapid hemodynamic changes^22,53,102^ so quick attentional shifts are likely to be cancelled out by the main signal.

Once participants’ behavioral patterns were video-coded, the subsequent steps involved identifying sustained attention moments within these observations, aligning the behavioral observations with the fNIRS recordings, and selecting an appropriate baseline for subsequent fNIRS analysis.

We reasoned that if attention follows the temporal sequence identified in previous heart rate attention literature^46^, the sustained attention window of interest would fall between 5 and 15 s after the infant orients towards (and engages with) a new stimulus. As established using heart rate recordings, this period of several seconds of sustained attention would include different phases, namely, pre-attention, attention orienting, and sustained attention phases (Fig. 1). Instances where infants attended to an object for more than 8 s (as defined by our video-coding analysis) were identified and marked as sustained attention events, consistent with previous ECG work showing that sustained attention typically begins 5 s after the onset of a look. Although sustained attention moments typically last around 10 to 15 s following the onset of a look, all observations lasting longer than 8 s were included in the analysis to maximize participant inclusion and ensure an adequate number of events per participant. The maximum duration was capped at 15 s to minimize intrasubject variability across observations.

To accurately correlate these phases with fNIRS data, the start of each behavioral observation was shifted by 3 s into the onset of a look. This adjustment was essential to maximize the likelihood of capturing brain activity related to the sustained attention phase as identified in previous studies^46^, while accounting for brain activity associated with attention orienting. Specifically, we reasoned that infants would enter the sustained attention window approximately 5 s after beginning to engage with a stimulus. Brain activity related to sustained attention would then be observable in the fNIRS data approximately 5–8 s after the initiation of sustained attention, with the peak occurring after 10–15 s, reflecting the hemodynamic response delay typical in fNIRS studies, where stimulus-evoked increases in HbO are generally detectable 5–8 s after stimulus presentation^67,83^. Consistent with this hypothesis, the first 3 s following the start of a sustained attention event, as defined behaviorally (i.e., when the infant clearly focused on the toy, indicated by their eyes and head being oriented towards the stimulus), were designated as the baseline to capture the pre-attention and attention-orienting phases. In addition, sustained attention events with interstimulus intervals shorter than 5 s were excluded from fNIRS data analysis to prevent overlap between the delayed fNIRS response during sustained attention and the subsequent baseline. In other words, rather than attempting to identify moments of no attention, different attentional states were used to define an appropriate baseline. Specifically, the initiation of engagement with a new stimulus (corresponding to the pre-attention and orienting phases in attention-heart-rate literature) was chosen as an active baseline^22,80–82^ for subsequent sustained attention-related fNIRS activity. A visual representation of the alignment between the behavioral observations and the expected timing of the hemodynamic response is provided in Fig. 1.

To further validate the accuracy of the selected time window, fNIRS data analysis was conducted by anticipating and postponing the start times of the sustained attention blocks by three seconds, and the temporal unfolding of the hemodynamic response (HR) within each channel was examined. Given the typical delay in the HR^67,83^, we reasoned that if the start of the sustained attention blocks is aligned with the onset of the look, brain activity related to sustained attention may extend beyond the fNIRS window of interest. Conversely, if the start of the sustained attention block is shifted further into the look, this postponement would likely enhance the specificity of capturing brain activity related to sustained attention, due to the typical delay in the hemodynamic response observed in fNIRS studies.

Lastly, we conducted an additional analysis in which the start of each attention window was randomized (± 15–30 s) to ensure that our findings were not dependent on a specific time window definition. We also implemented a cluster-based permutation analysis to assess the spatial robustness of the observed effects.

## Data Availability

The datasets generated during and/or analyzed during the current study are available from the corresponding author on reasonable request.
